# The effect of concurrent neural injuries on hemorrhage

**DOI:** 10.3389/fneur.2026.1848234

**Published:** 2026-07-08

**Authors:** Alyssa Trevino, Kayli N. Colpitts, Victoria Balentine, James W. Grau

**Affiliations:** 1 Psychological and Brain Sciences, Texas A&M University, College Station, TX, United States; 2 Institute for Neuroscience, Texas A&M University, College Station, TX, United States

**Keywords:** hemorrhage, locomotor recovery, pain, polytrauma, secondary injury, spinal cord injury, traumatic brain injury

## Abstract

**Objective:**

Spinal cord injury (SCI) is often accompanied by additional tissue damage (polytrauma) that amplifies inflammation and activates pain pathways. The latter has been studied by engaging nociceptive fibers using electrical stimulation or capsaicin caudal to a thoracic SCI. Nociceptive stimulation 1 day after SCI increases hemorrhage, amplifying secondary tissue loss. Noxious stimulation also promotes hemorrhage after a traumatic brain injury (TBI). A common form of polytrauma after SCI involves a TBI. The current study examines whether a concurrent TBI promotes hemorrhage after SCI. This also allowed us to evaluate whether a concurrent SCI promotes brain hemorrhage after TBI.

**Methods:**

Animals received a thoracic SCI and a concurrent brain surgery (anesthesia alone, craniectomy, or TBI). Other animals received a TBI to the frontal region and a concurrent spinal surgery (anesthesia alone, laminectomy, or SCI). Tissue was collected 24 h later, sectioned, and the extent of brain/spinal cord hemorrhage was quantified. Sham controls were included to verify a remote injury (SCI/TBI) does not induce hemorrhage in the absence of local neural damage.

**Results:**

A concurrent TBI with a SCI amplified hemorrhage in the spinal cord. A craniectomy had an intermediate effect on hemorrhage. Additionally, concurrent SCI with a TBI increased hemorrhage in the brain with a more modest effect.

**Conclusion:**

The results provide a link between hemorrhage development and concurrent neural injuries, with greater hemorrhage observed after SCI in animals with a concurrent TBI. SCI modestly impacted hemorrhage after TBI. These results provide a basis to further investigate the mechanisms responsible for interactions between multiple neurotraumatic injuries.

## 1 Introduction

Spinal cord injury (SCI) patients often suffer a concurrent traumatic brain injury (TBI), the occurrence ranging from 4 to 59% (1, 2). This variability likely reflects differences in detection and classification, as mild TBIs are often underreported until symptoms emerge. While recent work has explored the consequences of pain and inflammation following neural injury (3, 4), relatively few studies have examined the effects of concurrent neural injuries in a preclinical model.

Building on this gap, emerging evidence suggests that neural processes outside the primary site of injury can influence tissue loss and recovery following SCI. Evidence for this comes from preclinical work examining the effect of engaging pain (nociceptive) fibers caudal to a contusive SCI in rats using either electrical stimulation or capsaicin. When applied a day after injury, both forms of nociceptive stimulation foster hemorrhage at the site of injury, thereby increasing secondary tissue loss and undermining long-term recovery in both male and female animals (3–5). Interestingly, inhibiting communication with the brain, by slowly infusing an anesthetic rostral to injury, blocks the adverse effect of noxious stimulation (6, 28). So too does inducing a state of general anesthesia using pentobarbital or isoflurane (7). The implication is that rostral neural systems, engaged by surviving nociceptive fibers, drive secondary tissue loss after SCI. Conversely, research has shown that treatments that sensitize nociceptive systems within the spinal cord (e.g., application of capsaicin to a hind paw; (4)) fosters hemorrhage after TBI, and this effect too is blocked by general anesthesia (8). Further, evidence suggests that the regulation of nociceptive activity in the spinal cord, and the development of neural over-excitation, is modulated by TBI (9, 10). Taken together, these findings suggest that tissue loss and hemorrhage at the site of injury is impacted by nociceptive/neural activity in other regions of the central nervous system.

Given the minimal preclinical work focusing on concurrent neurotrauma, the current study examines whether a concurrent brain injury affects hemorrhage development after SCI. The experimental design also allowed us to examine whether a remote injury (SCI) fosters hemorrhage after a cortical impact to the frontal brain region. This preliminary study shows that concurrent neural injury fosters hemorrhage in other regions of the central nervous system (CNS), laying the groundwork for future studies examining the mechanism and consequences of this form of polytrauma.

## 2 Materials and methods

### 2.1 Animals

Adult male Sprague Dawley rats were obtained from Envigo (Houston, TX). Animals were dual housed with a 12-h light/dark cycle and *ad libitum* access to food and water. All procedures were conducted during the light phase. The animals were housed for at least 7 days prior to being acclimated to locomotor assessment procedures on three separate days. Animals were randomly assigned to treatment conditions. All animals were included except those showing neural damage from the sham surgery. The experiments were approved by the Institutional Animal Care and Use Committee at Texas A&M University (TAMU). All procedures conformed to the standards for care and use of laboratory animals set forth by the National Institute of Health (NIH). Every effort was made to reduce the suffering of animals within experiments and limit the number used.

### 2.2 Surgical procedures

#### 2.2.1 Spinal cord contusion surgery

Animals in the SCI condition received a moderate contusion to the spinal cord at T11 using the New York University (NYU) Multicenter Animal Spinal Cord Injury Study (MASCIS) device. Rats were anesthetized with 5% isoflurane and medical oxygen, then maintained at 2–3% during surgery. They were shaved and the surgical site was cleaned with betadine and isopropyl alcohol. An incision was made longitudinally centered over T11 then two 6 cm longitudinal incisions were made on each side of the spinal column. Following the clearing of tissue, a laminectomy was performed at T11 to expose the spinal cord. The animal was secured in the MASCIS device, and the impactor was centered on the spinal cord. For the contusion condition the 10 g impactor was dropped from a 12.5 mm height with a 3 s dwell time. The incision was then closed with Michel clips. For the sham condition, animals received the same procedure with the exception of the impact. For the anesthesia condition, animals were anesthetized with isoflurane (5% induction, 2–3% maintenance) for the average duration of the contusion surgery.

#### 2.2.2 Traumatic brain injury surgery

Animals in the TBI condition received a moderate TBI to the right frontal region with a controlled cortical impact device (Leica Impact One). Animals were anesthetized with 5% isoflurane and medical oxygen, then maintained at 2–3% during surgery. Animals were placed in a stereotaxic frame then were shaved and cleaned with betadine and isopropyl alcohol. A 5 mm craniectomy was performed over the right frontal region. The dura was examined before and after injury to verify no dural tear occurred. The 2.0 mm impactor tip was centered before impacting at a velocity of 4 m/s with a 3 mm deformation depth. An adhesive cap was secured over the craniectomy to protect the underlying tissue. The incision was closed with super glue. For the sham procedure, animals received the same treatment with the exception of the impact. For the anesthesia condition, animals were anesthetized with isoflurane (5% induction, 2–3% maintenance) for the average duration of the TBI surgery.

#### 2.2.3 Post-surgical care

Following the second surgery, animals received an intraperitoneal (i.p.) injection of penicillin (100,000 units/kg) to prevent infection and 3 mL of 0.9% saline to compensate for fluid loss during the surgeries. Animals recovered for 24 h in a temperature-controlled 12-h light–dark cycled room with access to food and water *ad libitum*. No analgesics were used because nociceptive activity contribute to the effect of polytrauma (5, 11, 12). Further, treatment with anesthesia can impact injury outcomes, both functionally and physiologically (13, 14). Bladders were manually expressed at least twice daily for animals that received a SCI. Laminectomy and spinal anesthesia animals were handled in a similar fashion to control for handling stress.

### 2.3 Locomotor performance

Locomotor function was assessed using the scoring system developed by Basso, Beattie, and Bresnahan (BBB; 29) while animals explored an open field. Locomotor function was assessed 1 day after injury.

### 2.4 Euthanasia and tissue collection

Animals were euthanized with a lethal dose of pentobarbital (100 mg/kg) immediately following locomotor assessment. Prior to perfusion, spleens were removed and weighed. Because spleen weight covaries with animal weight, adjusted spleen weights (spleen weight divided by before surgery animal weight multiplied by 1,000) are reported. Animals were perfused with 0.9% saline followed by 4% paraformaldehyde. Brains and spinal cords were collected and stored at 4 °C in 4% paraformaldehyde for 72 h then moved to a 30% sucrose solution. The tissue was then embedded in Tissue-Tek O. C. T for histological examination. A cryostat was used to obtain 20 μm coronal brain sections and transverse spinal cord sections. Sections were mounted on glass slides and stored at −20 °C.

### 2.5 Assessment of hemorrhage

**Figure 1:**
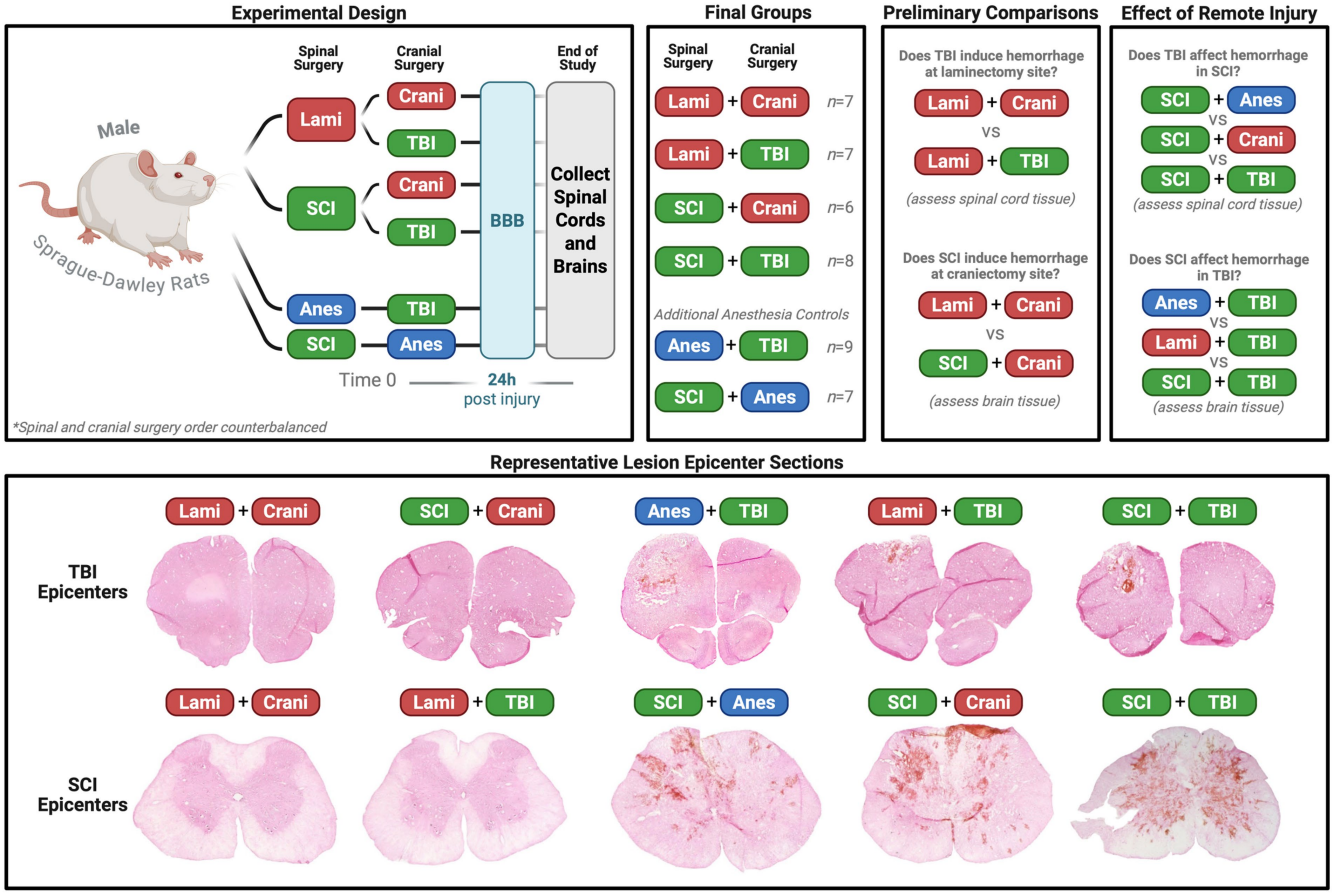
The experimental design, comparisons, and representative sections are illustrated. Animals received a SCI or laminectomy (Lami) with a TBI or craniectomy (Crani). Anesthesia (Anes) alone conditions were included to determine whether a remote neural injury had an effect in the absence of surgery at the target region and to assess the effect of a sham surgery. Representative injury epicenter sections are shown by group.

Tissue sections were stained with hematoxylin and eosin (H&E) to estimate the hemorrhage area of each section as described in (5). Slides were set out to dry at room temperature for approximately 3 h before staining began. Slides were first rinsed in distilled water for 45 s, then incubated in Mayer’s hematoxylin (Sigma-Aldrich, Ref# MHS32) for 4 to 5 min. Slides were rinsed in distilled water then dipped in acid alcohol (1% hydrochloric acid (HCl) and 70% ethanol (EtOH)) for 45 s. Slides were rinsed in distilled water then dipped in Scott’s tap water (Electron Microscopy Sciences, Cat# 26070–06) for 1 min. Finally, slides were incubated in eosin Y (Sigma-Aldrich, Ref# HT110316) for 15–20 s and dehydrated with ethanol and xylene before mounting with Permount (Electron Microscopy Sciences, Cat#17986–01). Sections were imaged using light microscopy at 2x magnification using 4–8 images per brain section and 1 image per section for the cords. Brain sections were stitched using the Grid/Collection stitching plugin in the Fiji software (15). Total area and total hemorrhagic area were color thresholded and manually corrected by a blinded observer. Examples of sections with the hemorrhagic area traced are provided in the Supplementary material. Assessment of hemorrhage in the brain was only done in the right lobe due to the focal nature of the injury. Injury epicenter for both brains and spinal cords were determined by the section with the highest hemorrhagic area percentage. Representative injury epicenters for each group are provided in Figure 1. Three additional sections taken at 500 microns intervals both rostral and caudal to the epicenter were selected and quantified.

### 2.6 Statistics

All data are presented as mean +/− SEM and were analyzed in jamovi using an analysis of variance (ANOVA). Non-parametric group comparisons were performed using the Kruskal-Wallis test, followed by pairwise comparisons using the Dwass-Steel-Critchlow-Fligner test. The criterion of *p* < 0.05 was set as the threshold for statistical significance.

### 2.7 Experimental designs

All animals (*N* = 44) received a spinal surgery condition [anesthesia alone (Anes), laminectomy (Lami), or (SCI)] and a brain surgery condition [anesthesia alone (Anes), craniectomy (Crani), or (TBI)]. Surgeries were conducted in immediate succession, with order counterbalanced across conditions. To minimize animal use, groups with anesthesia for both procedures or anesthesia with a craniectomy or laminectomy were not included as hemorrhage would not be expected in these groups. After 24 h, animals’ locomotor function was assessed. Animals were then euthanized, perfused, and tissue was collected for further histological analyses. The extent of brain and spinal cord tissue hemorrhage was quantified. The experimental design and final group sample sizes are shown in Figure 1. While the observation period is brief, previous work has demonstrated that noxious stimulation induces an increase in hemorrhage within hours and related long-term outcomes such as reduced locomotor function and increased lesion sizes that this produces (3–5, 11, 12).

## 3 Results

### 3.1 A remote neural injury does not produce signs of hemorrhage in sham operated animals

We sought to examine whether concurrent SCI and TBI have a synergistic effect that amplifies the extent of damage at both sites. Before assessing this issue, we examined tissue from sham operated animals to confirm that a remote injury has no effect in the absence of a neural injury at the other site (Figure 1, “Preliminary Comparisons”).

We performed histological analyses of spinal cord tissue from animals that had received either craniectomy or a TBI. Animals that had received a sham surgery at both sites exhibited virtually no signs of spinal cord damage (mean = 0.00113%/section), and this was unaffected by a concurrent TBI (mean = 0.00112%/section). An ANOVA confirmed that the Crani+Lami group and the TBI + Lami group differences were not statistically significant (*F*_(1, 12)_ < 1.00, *p* > 0.05).

Likewise, histological analyses of brain sections from animals that had undergone a sham surgery at both sites found little evidence of damage (0.00045%/section). Few signs of brain hemorrhage were observed in animals that received a craniectomy combined with SCI (0.00063%/section). Again, an ANOVA confirmed that the Crani+Lami group and the Crani+SCI group differences were not significant (*F’s*_(1, 11)_ < 1.00, *p* > 0.05).

These observations confirm that our sham surgery did not induce hemorrhage, and a remote neural injury has no effect in the absence of a neural injury at the opposite site.

### 3.2 A concurrent TBI with a SCI increases hemorrhage at the site of SCI

**Figure 2:**
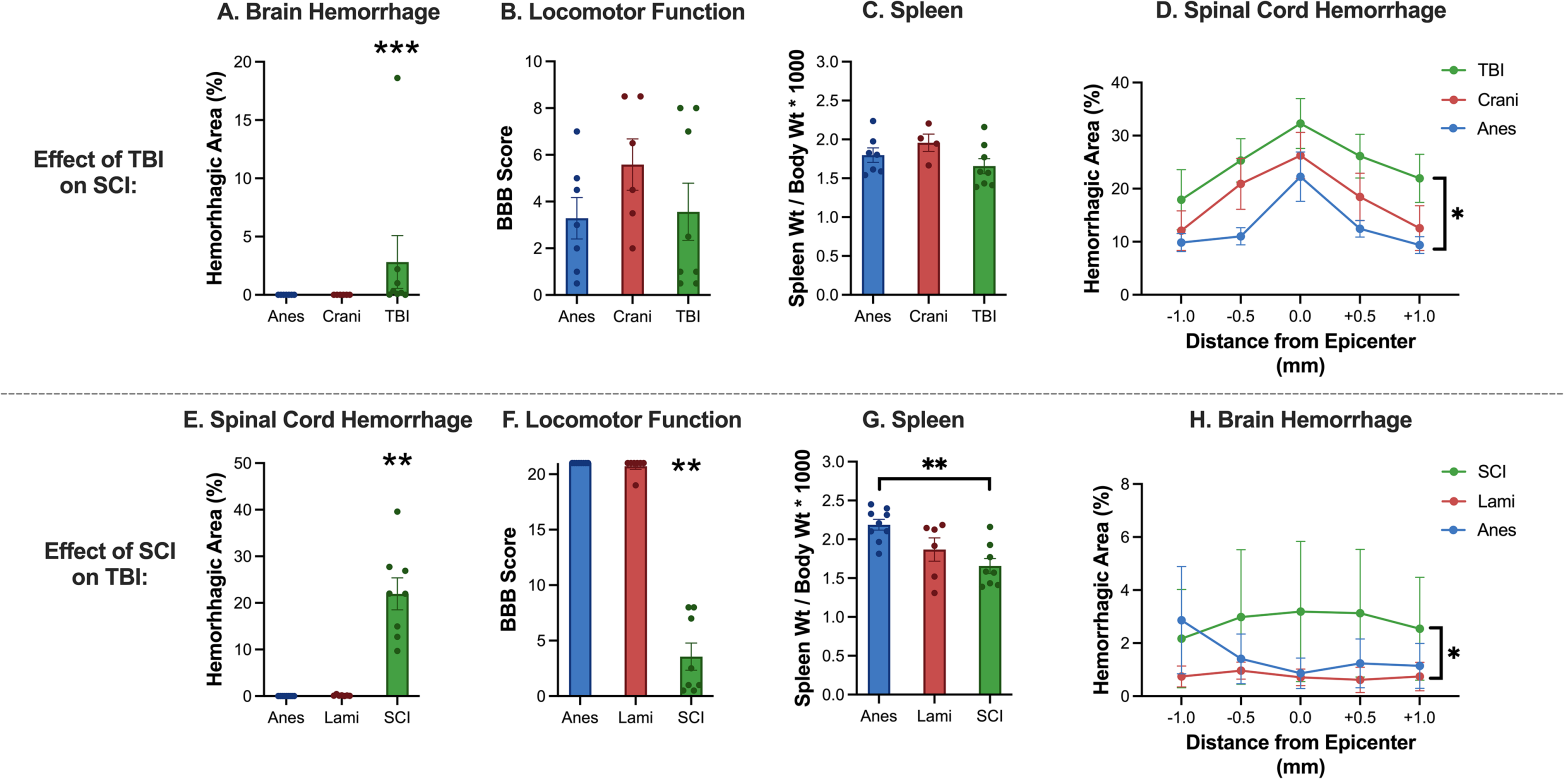
Panels **(A-D)** represent varied TBI conditions paired with SCI. **(A)** Percentage of hemorrhagic area on brain sections. **(B)** Locomotor performance. **(C)** Adjusted spleen weight. **(D)** Percentage of hemorrhagic area on spinal cord sections. Panels **(E–H)** represent varied SCI conditions paired with TBI. **(E)** Percentage of hemorrhagic area on spinal cord sections. **(F)** Locomotor performance. **(G)** Adjusted spleen weight. **(H)** Percentage hemorrhagic area on brain sections. All data are presented as mean +/− SEM. * *p* < 0.05; ** *p* < 0.01; *** *p* < 0.001. Sample size for all groups 6–8 animals, refer to Figure 1 for exact number of animals in each condition.

We then assessed whether remote tissue damage, due to a TBI or craniectomy surgery, affects the extent of hemorrhage after SCI (Figure 1, “Effect of Remote Injury”). As expected, the TBI increased brain hemorrhage relative to craniectomy and anesthesia controls (Figure 2A). Because these data exhibited some heteroscedasticity, a non-parametric test (Kruskal-Wallis) was used to compare the groups. This test confirmed that a TBI increased the area of brain hemorrhage (*X*^2^(2) = 17.60, *p* < 0.001). Pair-wise comparisons confirmed that the TBI group differed from the other two (*p* < 0.05).

As expected, animals that received a SCI exhibited poor locomotor performance (Figure 2B). This was not affected by a remote TBI (*F*_(2, 18)_ = 1.179, *p* > 0.05).

The remote injury did not affect spleen weight in animals that received a SCI (Figure 2C). An ANOVA confirmed that the group differences were not statistically significant (*F*_(2, 16)_ = 1.891, *p* > 0.05).

We then evaluated the extent of spinal cord hemorrhage in animals that had received a SCI and either anesthesia alone (Anes), a craniectomy surgery (Crani), or TBI (Figure 2D). Greater spinal cord hemorrhage was observed in animals that also received a TBI. An ANOVA confirmed that the groups differed (*F*_(2, 18)_ = 3.684, *p* < 0.05). *Post hoc* comparisons showed that animals that received a remote TBI differed from the anesthetized controls (*p* < 0.05). No other group comparison was significant (*p* > 0.05). The within subjects terms revealed a significant effect of location (*F*_(4, 72)_ = 10.955, *p* < 0.0001). This effect did not, however, interact with surgery condition (*F*_(8, 72)_ > 1.00, *p* > 0.05).

In summary, animals that received a TBI exhibited greater brain hemorrhage relative to craniectomy operated animals and anesthetized controls. A SCI led to worsened locomotor performance, which was unaffected by brain injury. Histological analyses of spinal cord tissue showed that a concurrent TBI increased hemorrhage at the site of SCI, relative to the anesthetized controls. Interestingly, the craniectomy (Crani) caused an intermediate effect on spinal cord hemorrhage.

### 3.3 A concurrent SCI with a TBI does not affect hemorrhage at the site of TBI

Next, we examined the effect of a concurrent SCI, or remote tissue damage (Crani) on brain hemorrhage in animals that received a TBI (Figure 1, “Effect of Remote Injury”). As expected, SCI increased the area of hemorrhage in the spinal cord tissue relative to animals that received a laminectomy or anesthesia alone (Anes; Figure 2E). An ANOVA confirmed that there was a significant effect of SCI (*F*_(2, 21)_ = 44.910, *p* < 0.0001). *Post hoc* comparisons showed that the SCI group differed from the other two (*p* < 0.05).

SCI disrupted locomotor performance relative to animals that had received a laminectomy or anesthesia alone (Figure 2F). An ANOVA confirmed that the groups differed (*F*_(2, 21)_ > 191.54, *p* < 0.0001). *Post hoc* comparisons showed that the SCI group differed from the other two (*p* < 0.05).

A remote injury reduced spleen weight (Figure 2G). An ANOVA confirmed that there was a significant effect of surgery condition (*F*_(2, 20)_ = 7.786, *p* < 0.005). *Post-hoc* comparisons showed that the SCI group differed from the anesthetized controls (Anes; *p* < 0.05). No other comparisons were significant (*p* > 0.05).

We then assessed the extent of brain damage in animals that received a TBI and a concurrent SCI, laminectomy (Lami), or anesthesia alone (Anes; Figure 2H). Again, the greatest hemorrhage was observed in the group that received a remote neural injury (SCI). While the main effect of treatment condition was not statistically significant (*F*_(2, 21)_ < 1.0, *p* > 0.05), the interaction between SCI condition and location approached significance (*F*_(8, 84)_ = 1.907, *p* = 0.0695). The main effect of location was not significant, (*F*_(4,84)_ < 1.0, *p* > 0.05). To further explore the SCI condition by location interaction, additional analyses were performed, comparing the SCI treated group to each of the control conditions. These analyses revealed a significant treatment by region interaction when the SCI group was compared to the anesthetized controls (*F*_(4, 60)_ = 2.693, *p* = 0.0393), but not the Crani+SCI group (*F*_(4, 52)_ = 1.032, *p* > 0.05).

In summary, SCI induced significant hemorrhage at the spinal cord lesion, impaired locomotor function, and produced a drop in spleen weight. SCI increased the extent of brain hemorrhage observed after TBI (relative to the anesthetized controls) and the magnitude of this effect varied along the caudal-rostral axis. Again, remote tissue damage (Lami+TBI) produced an intermediate effect.

## 4 Discussion

A large proportion of SCIs are accompanied by a concurrent neural injury or additional tissue damage (polytrauma). Our past work has modeled polytrauma using cutaneous stimuli that engage nociceptive fibers following a neural injury. Here we extended this work by examining the effect a concurrent TBI has on a SCI, a frequent clinical occurrence.

We first established that, in the absence of local neural injury, a remote insult (SCI or TBI) does not independently induce hemorrhage at the target site. It was then shown that a TBI amplified SCI hemorrhage. Remote tissue damage alone (craniectomy) had an intermediate effect. A similar, but more modest, effect was seen when assessing brain hemorrhage. A concurrent SCI modestly increased hemorrhage after TBI and the magnitude of this effect grew along the caudal-rostral axis.

Although the incidence of concurrent neural injury is high, the phenomenon has been relatively understudied. To our knowledge, this phenomenon has only been studied one other time. Inoue et al. (16) examined the effects that a concurrent SCI and TBI had on forelimb function. They found that a concurrent injury produced significant reductions in forelimb capabilities in animals with SCI + contralateral TBI compared to those receiving a SCI alone. They also found that an ipsilateral TBI + SCI restored paw placement performance to sham levels (16). Our work aligns with these results, showing that a concurrent TBI has adverse effects on a SCI.

The overall pattern of results is also consistent with prior work indicating that neural activity in other regions of the central nervous system, and noxious stimulation, can foster hemorrhage after SCI or TBI (4, 7, 8). Likewise, there is ample evidence that a neural injury can influence nociceptive systems; both SCI and TBI have been shown to foster the development of chronic pain (17–19). Given these observations, pretreatment with an analgesic (morphine) should have a protective effect. As predicted, pre-emptive analgesia attenuates pain-induced hemorrhage after TBI (8). However, morphine undermined locomotor function and increased mortality in animals that experienced a SCI and noxious stimulation (20, 21), which raises concerns regarding the widespread use of opiates to treat pain after SCI. As noted above, general anesthesia has been shown to attenuate nociception-induced hemorrhage after SCI or TBI. This raises the possibility that suppressing supraspinal activity through pharmacological sedation may reduce secondary tissue loss following concurrent neural injury. Ongoing studies are examining whether sustained sedation confers similar protective effects (22). Based on prior work, nociceptive signaling could potentially be fueling the effects observed in the present study, however systemic processes could also play a role.

Other work has shown that systemic processes and inflammation can contribute to tissue loss after injury. Both SCI and TBI induce a systemic inflammatory response, leading to an increase in pro-inflammatory cytokines, which is exacerbated with polytrauma (4, 23–25). Further, treatments that induce hemorrhage after SCI have been shown to drive a rise in systolic blood pressure and reduce spleen weight, suggesting systemic stress (26). Conversely, disrupting the pituitary–adrenal axis (*via* adrenalectomy) attenuates nociception-induced hemorrhage after SCI (27). In contrast, nociception-induced hemorrhage after TBI was not accompanied by a rise in blood pressure or a change in spleen weight. Further, nociceptive-induced tissue loss following TBI was shown to depend upon the site of stimulation, where capsaicin applied to the contralateral, but not ipsilateral paw, exacerbated tissue loss (8). Because systemic processes (e.g., inflammation) should develop independent of the site of stimulation, these observations suggest that the effect of noxious stimulation on brain hemorrhage is tied to the engagement of pain fibers (which decussate after they enter the spinal cord).

This preliminary study provides the foundation for future work examining the consequences of concurrent neural injuries. A limitation of the current study is that it was conducted exclusively in male animals, which aligns with the higher incidence of polytrauma observed in men. Future studies should evaluate whether these effects generalize to females and explore sex-dependent mechanisms. Additionally, this study is limited to an acute period and only assessed moderate injury severities. Further work is needed to assess the longitudinal consequences and evaluate the effect of different injury severities. Additional behavioral and cellular analyses are also suggested to uncover the processes that contribute to tissue loss and the effect of pain. Finally, studies are needed to evaluate treatments to mitigate secondary tissue loss in the context of concurrent neural injury and the cellular processes that underlie these effects.

## Data Availability

The raw data supporting the conclusions of this article will be made available by the authors, without undue reservation.
