# Supplementary figures and images for: The effect of concurrent neural injuries on hemorrhage
Source: Front Neurol. 2026 Jul 8;17:1848234. doi: 10.3389/fneur.2026.1848234 (PMC13388129; doi:10.3389/fneur.2026.1848234)

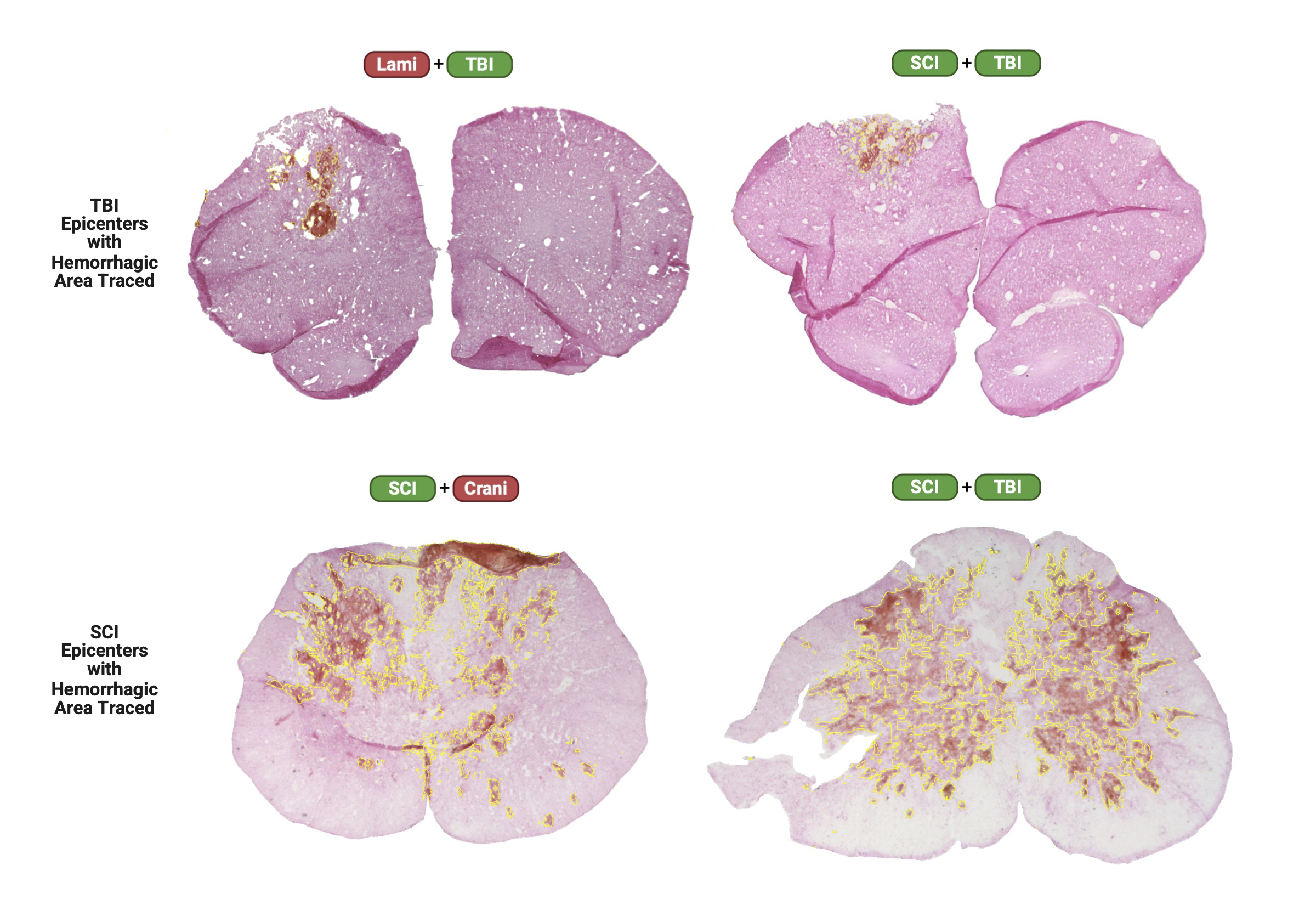

Supplement: Supplementary file 1 [file Image_1.tiff]
